# Analysis of the Academic and Professional Profiles of Dental Educators Promoting Courses at Instagram University

**DOI:** 10.1111/eje.70159

**Published:** 2026-04-20

**Authors:** Laís Albuquerque Fernandes, Luiz Augusto Rodrigues dos Santos, Erick Elienai Reis Araújo, Alexandre Hugo Llanos, Ricardo Grillo, Mariana Aparecida Brozoski

**Affiliations:** ^1^ Department of Oral and Maxillofacial Surgery, School of Dentistry University of São Paulo São Paulo Brazil; ^2^ School of Dentistry University of São Paulo São Paulo Brazil; ^3^ Department of Stomatology, School of Dentistry University of São Paulo São Paulo Brazil

**Keywords:** certification, digital media, educational status, social media

## Abstract

**Background:**

This study evaluated the current landscape of Brazilian dental education by analysing Instagram posts from professionals promoting dentistry courses. The objective was to ascertain the relationship between their qualification levels and digital visibility.

**Methodology:**

A retrospective analysis was conducted on 480 publicly available Instagram posts, tagged with specific dentistry course‐related hashtags, across 10 specialties with high numbers of registered professionals. Posts were screened for relevance, excluding non‐educational or purely promotional content.

**Results:**

The sample comprised 50 professionals per specialty, with a mean time since undergraduate graduation of 20.91 years. Orofacial Harmonization (HOF) emerged as a specialty with significantly less experienced professionals compared to Dental Prosthesis, Orthodontics, and Implantology. Similarly, Oral and Maxillofacial Surgery (OMFS) demonstrated shorter time since graduation compared to Dental Prosthesis, Orthodontics, and Implantology. Radiology exhibited the highest proportion of specialists, while Periodontics, Prosthodontics, and Radiology showed the highest proportion of professors with a master's degree. Radiology and Paediatric Dentistry led in the proportion of PhD holders, indicating a stronger academic background. Conversely, HOF had the lowest proportion of professors across all academic levels (Specialist, Master, and PhD), notably being the only specialty without independent PhDs. Restorative Dentistry also showed lower academic qualifications.

**Conclusions:**

This study highlights the complex interplay of visibility, academic success, and digital presence in Brazilian dental education, which varies significantly across specialties. This complexity underscores the need for customized strategies for workforce development. While comprehensive, the study's limitations suggest future research should include longitudinal studies of career paths, qualitative analyses of specialty selection, and platform‐specific engagement data to better understand this evolving healthcare environment.

## 1 Introduction

Social media platforms have transformed knowledge dissemination in dentistry. Instagram, with one billion monthly active users by 2020 [1], has become a key tool for professional communication. Originally a photo‐sharing application, its mobile‐friendly interface facilitates widespread engagement among both practitioners and the public.

Dental professionals increasingly use Instagram for education and marketing. Evidence suggests a growing trend of academic accounts leveraging the platform for student recruitment and patient outreach [2]. In this context, digital pedagogy encompasses various educational functions that warrant detailed examination. First, student recruitment involves the use of visual content to attract prospective applicants to dental programs by showcasing campus facilities, student experiences, and career opportunities. Second, course promotion refers to the marketing of continuing education courses, specializations, and workshops designed for practicing dentists seeking professional development. Third, patient education encompasses the dissemination of accessible information regarding oral hygiene, treatment procedures, and preventive care, directly engaging with the public to improve health literacy. Fourth, oral health worker training involves the use of Instagram to share instructional content targeted at dental assistants, hygienists, and community health workers, supporting workforce development in diverse clinical settings. When used ethically, Instagram may bridge the gap between theoretical instruction and clinical practice across these diverse applications [3, 4].

Brazil represents a critical market for this phenomenon, ranking third globally in Instagram usage with 140 million active users [5]. The country's strong emphasis on medical and dental education [6, 7] makes it an ideal setting for analysing digital pedagogy. Over 50% of Instagram users follow healthcare professionals [8], indicating significant potential for educational outreach across the four domains of student recruitment, course promotion, patient education, and oral health worker training. The Brazilian context thus offers a valuable opportunity to examine how dental educators leverage social media to fulfil these pedagogical functions.

Accordingly, this study aims to examine the relationship between dental educators' academic qualifications and their digital visibility on Instagram. The analysis focuses on course‐promoting content to assess how expertise influences audience engagement. Findings may inform best practices for social media use in dental education, providing guidance for educators seeking to optimize their digital presence for educational purposes while maintaining professional standards and ethical considerations.

## 2 Material and Methods

The study retrospectively analysed Instagram posts tagged with specific dentistry course‐related hashtags to assess marketing trends in dental education. Data were collected from publicly available posts containing the more usual hashtags over 10 specialties with more registered professionals. Posts were screened for relevance, with exclusions applied to non‐educational or purely promotional content unrelated to academic offerings.

Metadata from eligible posts, including captions, engagement metrics (followers), and associated user profiles, were extracted for further examination. Key variables of interest included academic discipline, academic level, professional experience of professors, and demographic data. Official sites outside social media were used to retrieve professional data.

### 2.1 Theoretical Framework and Assessment Parameters

This study was grounded in Bourdieu's field theory [9] to interpret how different forms of capital manifest across dental specialties. Within this framework, three distinct forms of capital were considered: academic capital (institutional affiliation and postgraduate degrees), cultural capital (specialization and professional experience), and emerging digital capital (social media visibility and follower engagement).

Academic qualifications were assessed using two primary parameters. First, academic affiliation was defined as formal employment or teaching position at a recognized higher education institution, verified through institutional websites and official dental school directories. Second, Lattes curriculum presence was verified through the publicly accessible Lattes Platform, Brazil's official academic database maintained by the National Council for Scientific and Technological Development (CNPq), which comprehensively documents scholarly production, academic trajectories, and research activities of Brazilian researchers. Postgraduate qualifications were categorized into three levels: Specialization (lato sensu postgraduate certificate), Master's degree (stricto sensu academic or professional master's), and PhD (doctoral degree). Professional experience was operationalized as years since undergraduate dental graduation, calculated from the year of first dental degree to 2024.

Digital visibility was quantified through Instagram follower counts, serving as a proxy for audience reach and influence within each specialty. Course‐promoting content was identified through specialty‐specific hashtags, and only posts explicitly advertising educational offerings were included in the analysis.

### 2.2 Statistical Analysis

Data organization and preliminary tabulation were performed using Microsoft Excel to ensure structured and clean datasets. Statistical analyses, including descriptive statistics and hypothesis testing, were carried out using RStudio. Pearson's correlation, Kruskal‐Wallis, and Dunn post hoc tests were used to evaluate differences in engagement metrics across multiple independent groups, such as academic disciplines or course levels. Where significant differences were detected, post hoc pairwise comparisons were conducted to identify specific group disparities. Results were considered significant if *p* < 0.05. Ethical considerations were adhered to by utilizing only publicly accessible data without extracting private or personally identifiable user information beyond aggregated metadata.

## 3 Results

A total of 480 posts from 10 different dental specialty courses were analysed. The sample size was standardized at 50 professionals per specialty (6.1% margin of error, *p* < 0.05), except for Dental Radiology which was reduced to 30 due to its limited population size. This adjustment ensured balanced representativeness across groups while preventing distortion from oversampling in smaller populations, while maintaining minimum sample requirements for other specialties. A summary of the search results is presented in Table 1.

**TABLE 1:** Characteristics of dental education posts on Instagram according to the number of registered specialists in Brazil.

Specialty	Usual hashtags	Registered specialists	Number of posts	Size sample (*n*)
Orthodontics	#especializaçãoortodontia	32 721	1000	50
Implandontics	#cursoimplantodontia	21 920	1000	50
Endodontics	#cursoendodontia	19 594	1000	50
Dental Prosthesis	#especializacaoprotese	14 327	1000	50
Periodontics	#cursoperiodontia	10 899	500	50
Odontopediatrics	#cursoodontopediatria	10 100	500	50
Oral and Maxillofacial Surgery (OMFS)	#cirurgiaoralmenor	7899	41 000	50
Dentistry	#cursodefacetas	7154	1000	50
Dental Radiology	#cursoradiologiaodontologica	5817	100	30
Orofacial Harmonization (HOF)	#cursoharmonizacaofacial	4709	22 000	50

Among Brazilian states, only three were not represented (AC, AP, and SE) (Figure 1). A strong correlation was observed between the number of posted courses and the state's total population (*ρ* = 0.9621, *p* < 0.001), as well as with the number of registered dentists in their respective states (*ρ* = 0.9848, *p* = 0.0018).

**FIGURE 1:**
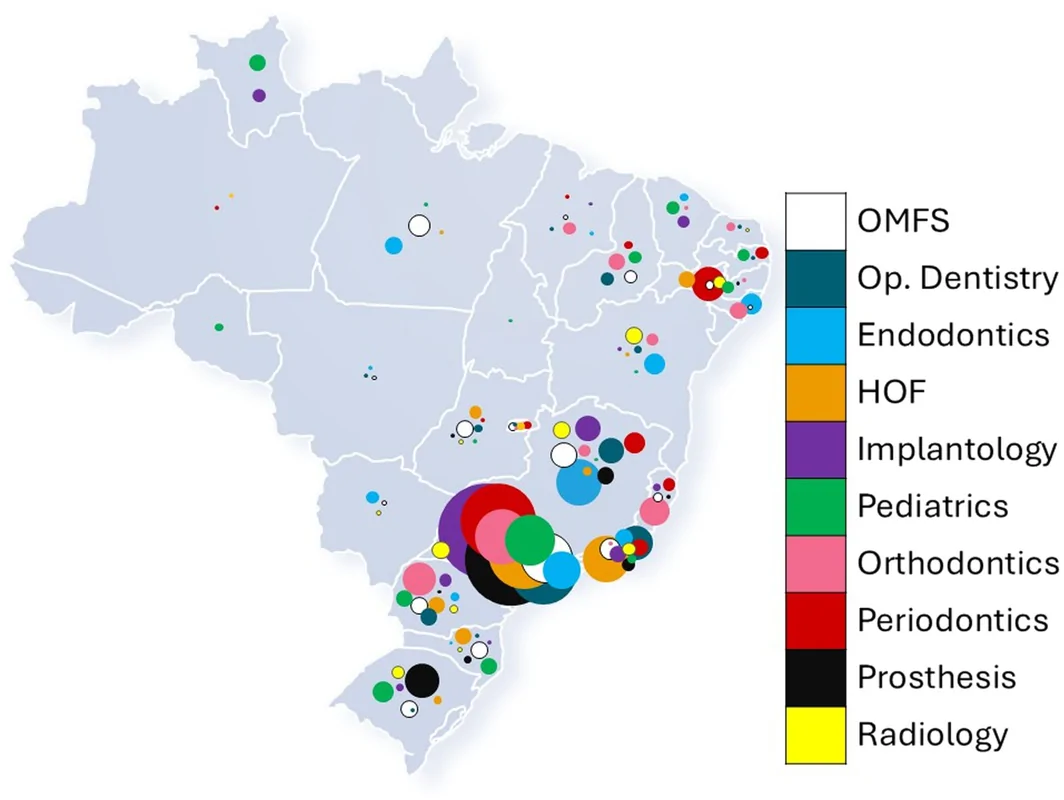
Distribution of professors promoting dentistry courses across Brazilian territories. Circle size corresponds to the number of courses posted per state. The map demonstrates the concentration of educational offerings in southeastern states (São Paulo, Rio de Janeiro, Minas Gerais), with three states (Acre, Amapá, Sergipe) showing no representation in the sampled posts.

The mean time since undergraduate graduation was 20.91 years [range: 2–57]. Standard deviation across specialties was 3.44. There were statistically significant differences in time since graduation among the various dental specialties (*H*(9) = 46.8; *p* < 0.0001). This overall analysis demonstrates that professional experience, measured in years since graduation, varies significantly among at least some of the specialist groups (Figure 2).

**FIGURE 2:**
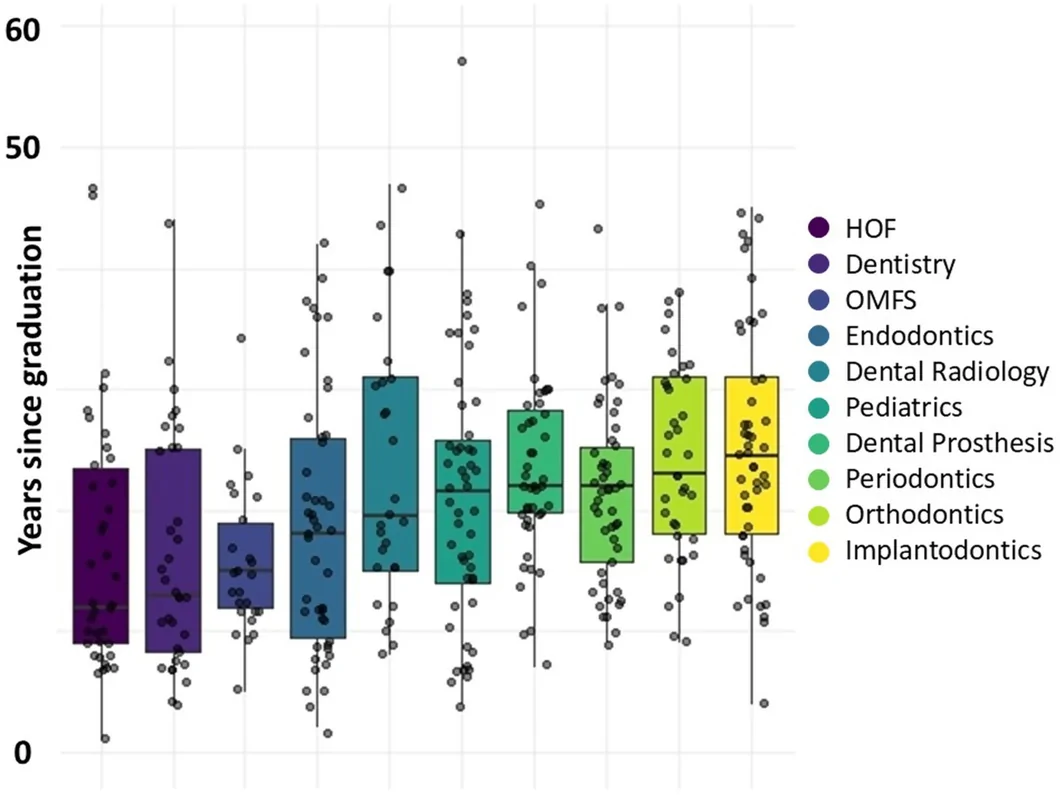
Distribution of professional experience among dental educators promoting courses on Instagram, measured as years since undergraduate dental graduation in Brazil. Box plots display median (horizontal line), interquartile range (box), and outliers (points) for each specialty.

Analyses demonstrated that HOF emerged as the specialty with significantly less experienced professionals compared to three other areas: Dental Prosthesis (*p* = 0.0128), Orthodontics (*p* = 0.00756) and Implantology (*p* = 0.00196). A similar pattern was observed for Operative Dentistry, which showed significantly shorter time since graduation than: Dental Prosthesis (*p* = 0.0180), Orthodontics (*p* = 0.0106) and Implantology (*p* = 0.00309). OMFS also demonstrated shorter time since graduation when compared to: Dental Prosthesis (*p* = 0.0204), Orthodontics (*p* = 0.0120) and Implantology (*p* = 0.00429).

There are statistically significant differences among specialties regarding professors' academic levels (*χ* ^2^ = 25.783, *p* = 0.002132), confirming disparities (Figure 3). Radiology has the highest proportion of specialists among its professors. Periodontics, Prosthodontics, and Radiology show the highest proportion of professors with a master's degree. Radiology and Paediatrics lead in the proportion of PhD holders, indicating a stronger academic background in these specialties. HOF has the lowest proportion of professors across all three academic levels (Esp, MSc, and PhD), being the only specialty without independent PhDs. Restorative Dentistry also ranks among the specialties with the lowest academic qualifications.

**FIGURE 3:**
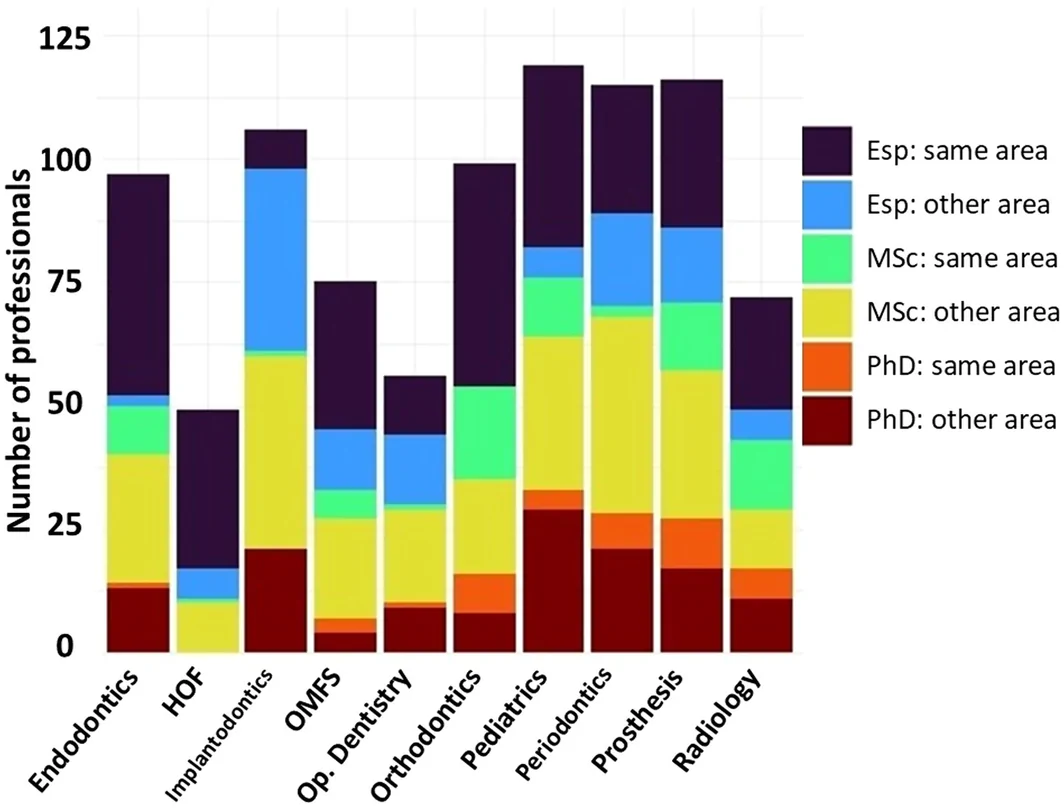
Distribution of postgraduate qualifications among dental educators promoting courses on Instagram, by Brazilian dental specialty. Stacked bar chart shows the proportion of educators holding Specialization (lato sensu certificate), Master's degree (stricto sensu), and PhD (doctoral degree) within each specialty.

Direct comparisons demonstrated that Periodontics, Paediatric Dentistry, and Radiology exhibited statistical superiority (*p* < 0.001) relative to other specialties, with high proportions of academic affiliation (62%, 60%, and 60%, respectively) and Lattes curriculum presence (94%, 82%, and 62%). In contrast, HOF was critically deficient, with 0% academic affiliation and only 34% Lattes presence (*p* < 0.001 in all comparisons), followed by Operative Dentistry with just 12% affiliation. An interesting pattern was observed in Endodontics, which demonstrated high Lattes prevalence (90%) alongside low academic affiliation (28%) (Figure 4).

**FIGURE 4:**
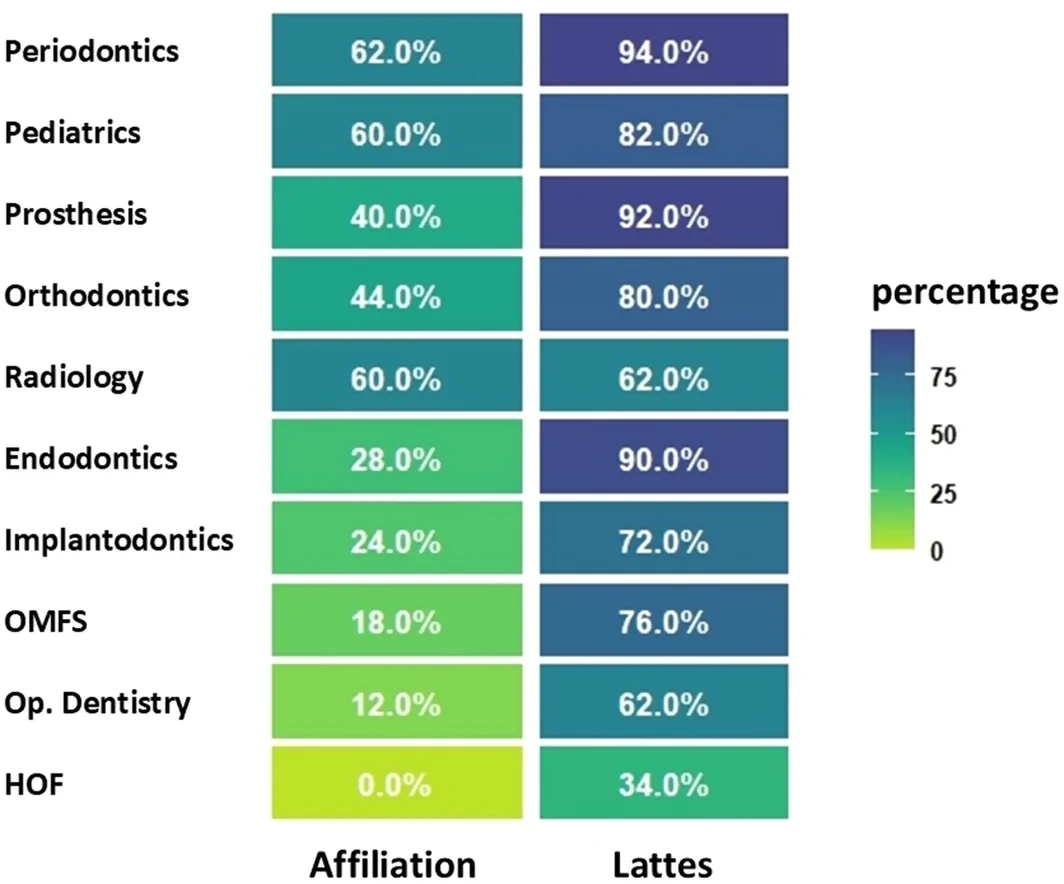
Comparison of academic affiliation and Lattes profile by specialty.

Radiology demonstrates the strongest negative correlation (*ρ* = −0.466, *p* < 0.05), indicating that each additional year since graduation corresponds to substantially fewer followers, with recent graduates averaging five times more followers than their senior counterparts. A similar but weaker negative association exists in Prosthodontics (*ρ* = −0.173), though this relationship does not reach conventional statistical significance.

Conversely, HOF shows the most robust positive correlation (*ρ* = 0.327, *p* < 0.10), where experienced professionals average 32 673 followers compared to 8192 for early‐career practitioners. Orthodontics displays a marginally positive relationship (*ρ* = 0.180), with mid‐career professionals maintaining moderately larger followings than recent graduates.

Most other specialties, including Endodontics (*ρ* = 0.047), OMFS (*ρ* = 0.058), and Paediatrics (*ρ =* 0.060), show negligible correlations, indicating follower counts remain relatively stable regardless of career duration. The ANOVA confirms significant between‐specialty variation in these patterns (*F* = 9.35, *p* < 0.001), accounting for approximately 18% of the observed variance in follower counts (Figure 5).

**FIGURE 5:**
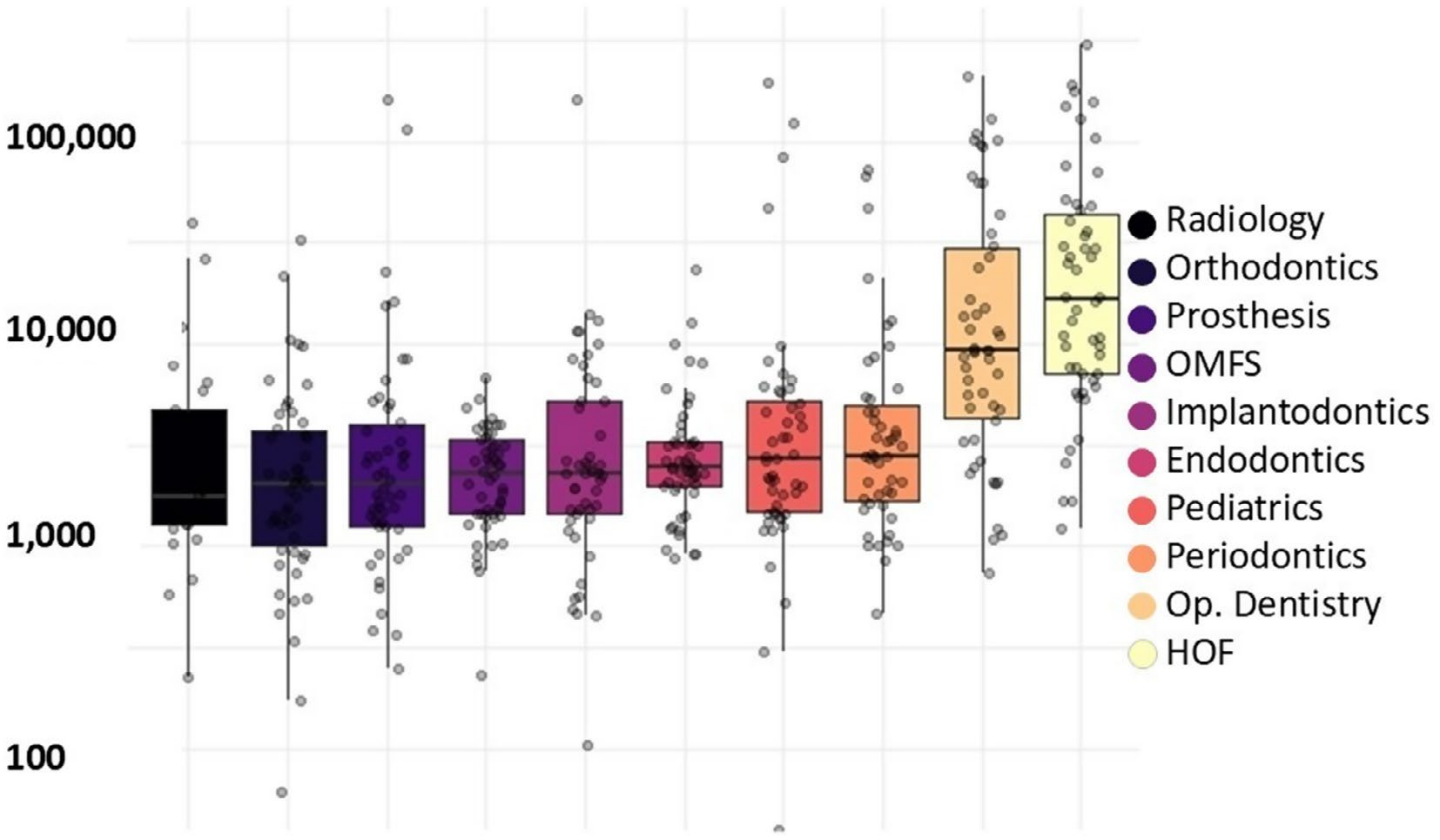
Number of followers distributed among specialties.

## 4 Discussion

New technologies are changing the role of students and professors [10, 11]. The near‐perfect correlations between course posting frequency and both population density (*ρ* = 0.9621, *p* < 0.001) and dentist concentration (*ρ* = 0.9848, *p* = 0.0018) suggest a highly market‐driven continuing education system. These robust correlations indicate that course offerings closely follow regional demand patterns, reflecting a responsive private education sector strategically targeting areas with higher professional density.

The significant variations in time since graduation across specialties (*H*(9) = 46.8, *p* < 0.0001) reveal fundamental differences in career trajectories. HOF professionals demonstrate significantly fewer years of experience than traditional specialties like Prosthodontics and Orthodontics (*p* < 0.05 for all comparisons), suggesting this emerging field attracts younger practitioners, potentially due to its cosmetic focus and recent popularity surge [8, 12]. Similar patterns observed in Operative Dentistry and OMFS may reflect either generational shifts in specialty preference or alternative pathways to specialization. These temporal disparities have important implications for understanding clinical experience variations across dental services.

This analysis identifies profound generational differences in digital engagement strategies. Recent graduates show greater adaptation to emerging platforms like TikTok and Instagram Reels, while established professionals prioritize clinical and academic activities over social media presence [7, 13, 14]. This digital divide is particularly evident in Radiology, which shows the strongest negative correlation between years since graduation and follower count (*ρ* = −0.466, *p* < 0.05).

Academic qualification data (*χ* ^2^ = 25.783, *p* = 0.002) reveal striking specialty stratification. Periodontics, Paediatric Dentistry, and Radiology demonstrate the strongest academic profiles, with the highest proportions of both MSc and PhD holders. This contrasts sharply with HOF's complete absence of PhD‐level instructors. The Endodontics specialty presents an intriguing paradox, combining high Lattes platform participation (90%) with low institutional affiliation (28%), suggesting practitioners value scholarly activity independent of traditional academic engagement.

The specialty‐specific digital engagement patterns reflect deeper professional norms, with some fields requiring rapid digital adaptation while others maintain traditional emphasis on experiential authority [7, 15, 16, 17]. Implantology emerges as the specialty with the most experienced professionals, showing statistically significant differences (all *p* < 0.01) compared to other fields, suggesting either greater technical demands requiring extended training or market dynamics favouring established practitioners.

Three distinct specialty archetypes in social media influence patterns were identified: (1) digital‐native fields where recent graduates dominate; (2) experience‐dependent specialties where seniority predicts influence; and (3) neutral fields with experience‐agnostic digital presence. This tripartite division suggests fundamentally different professional reputation‐building mechanisms, with 18% of variance explained by specialty alone.

The findings carry significant theoretical implications when examined through the lens of Bourdieu's field theory, as outlined in our analytical framework, revealing how distinct forms of cultural capital manifest across dental specialties [9]. Traditional specialties (Prosthodontics, Orthodontics) maintain conventional academic capital, while emerging fields like HOF emphasize clinical‐technical capital [18]. Radiology appears to be developing digital capital as a professional asset. These insights suggest educational policies need differentiated residency requirements accounting for each specialty's unique knowledge base.

Our findings indicate professional development programs should incorporate specialty‐tailored digital training, recognizing varying needs between rapidly adapting fields and traditional reputation‐based specialties [10]. Market dynamics in continuing education must similarly evolve to accommodate specialty‐specific career timelines [19], as demonstrated by Implantology's experienced practitioners versus HOF's younger demographic.

## 5 Limitations

Several potential confounding factors warrant consideration when interpreting these findings. First, Instagram's algorithmic curation may systematically influence which educational content achieves visibility, potentially favouring certain specialties or presentation styles regardless of academic qualifications. The platform's engagement‐based ranking system could amplify content from aesthetically oriented specialties while diminishing reach for procedure‐based disciplines, independent of educator credentials. Second, temporal trends in platform adoption create cohort effects. Early Instagram adopters may have accumulated followers gradually over a decade, whereas recent entrants face saturated markets with different engagement dynamics. This temporal confounder could explain some variation in the experience‐follower correlations, as professionals who graduated recently but joined the platform early would have different visibility trajectories than those with similar experience but later adoption.

Regional variations in internet infrastructure, Instagram penetration, and dental market density introduce geographic confounding. Specialty‐specific content characteristics may confound the relationship between qualifications and visibility. Specialties producing highly visual content (e.g., before‐and‐after cosmetic procedures) may generate higher engagement irrespective of academic background, while specialties relying on conceptual knowledge face inherent disadvantages in image‐driven platforms. Follower count as a visibility metric may conflate genuine educational influence with entertainment value, personal branding, or non‐educational content appeal. These confounders suggest caution in attributing observed patterns solely to specialty‐specific professional norms. Future research should employ longitudinal designs tracking qualification acquisition alongside digital engagement, incorporate platform‐agnostic visibility metrics, and develop specialty‐weighted analytical models that account for inherent content visibility differences.

Regarding generalizability, while Brazil's unique combination of high Instagram penetration, robust postgraduate dental education infrastructure, and centralized academic credentialing system provides an ideal natural laboratory for this investigation, the fundamental relationships observed between academic qualifications and digital visibility likely extend to other countries with comparable social media adoption and dental education frameworks, though specialty‐specific patterns may vary according to local regulatory, cultural, and market conditions.

## 6 Conclusion

This study reveals a complex dental education landscape where professional visibility, academic achievement, and digital engagement interact in specialty‐dependent ways, demanding tailored workforce development strategies. While comprehensive, study limitations highlight the need for future longitudinal tracking of career trajectories, qualitative investigations of specialty choice motivations, and platform‐specific engagement analyses to further elucidate these dynamics in Brazil's evolving oral healthcare ecosystem.

## Author Contributions

Author(s) testify that all persons designated as authors qualify for authorship and have checked the article for plagiarism. Conceptualization and study design: L.A.F. and R.G. Data collecting: L.A.F., L.A.R.S., E.E.R.A., A.H.L. Analyses and data interpretation: L.A.F., L.A.R.S., E.E.R.A., A.H.L., R.G., M.A.B. Drafting: L.A.F., L.A.R.S., E.E.R.A., A.H.L., R.G., M.A.B. All authors have critically reviewed and approved the final draft and are responsible for the content and similarity index of the manuscript.

## Funding

The authors have nothing to report.

## Ethics Statement

The authors have nothing to report.

## Consent

The authors have nothing to report.

## Conflicts of Interest

The authors declare no conflicts of interest.

## Data Availability

The data that support the findings of this study are available from the corresponding author upon reasonable request.
